# Supplementary material for: Subtypes of Patients with Mild to Moderate Airflow Limitation as Predictors of Chronic Obstructive Pulmonary Disease Exacerbation
Source: J Clin Med. 2023 Oct 20;12(20):6643. doi: 10.3390/jcm12206643 (PMC10607211; doi:10.3390/jcm12206643)
Supplement: Supplementary file 1 [file jcm-12-06643-s001.zip › jcm-2642981-Supplement Table S1.pdf]

Supplement Table S1. Chest computed tomography (CT) findings

	Chronic bronchitis	Emphysema	Young smokers	Near-normal	p value
Sequelae of tuberculosis	6/121 (4.9%)	7/125 (5.6%)	6/117 (5.1%)	7/101 (6.9%)	0.923
Emphysema	41/121 (33.8%)	61/125 (48.8%)	56/117 (47.8%)	34/101 (33.6%)	0.017
Bronchiectasis	21/121 (17.3%)	5/125 (4.0%)	8/117 (6.8%)	15/101 (14.8%)	0.002

Chest CT was not performed in all patients.
