# Supplementary material for: Subtypes of Patients with Mild to Moderate Airflow Limitation as Predictors of Chronic Obstructive Pulmonary Disease Exacerbation
Source: J Clin Med. 2023 Oct 20;12(20):6643. doi: 10.3390/jcm12206643 (PMC10607211; doi:10.3390/jcm12206643)
Supplement: Supplementary file 1 [file jcm-12-06643-s001.zip › jcm-2642981-Supplement Table S2.pdf]

Supplement Table S2. Univariate logistic regression analysis of risk factors associated with exacerbation of COPD

	Univariate analysis		
	OR	95% CI	*p*-value
Cluster			
Near-normal	1.000		
Young smokers	1.326	0.884-1.999	0.174
Emphysema	1.929	1.294-2.896	0.001
Chronic bronchitis	1.395	0.923-2.118	0.116
Age	1.012	0.994-1.031	0.203
Female	1.414	0.852-2.313	0.173
Body mass index	0.976	0.935-1.019	0.273
Smoking status			
Never	1.000		
Former	0.996	0.602-1.688	0.987
Current	1.118	0.648-1.969	0.692
Smoke pack-years	1.004	0.999-1.010	0.150
Past history of asthma diagnosis	1.546	1.143-2.089	0.005
FEV_1 % predicted	0.980	0.968-0.991	0.001
FVC % predicted	0.984	0.974-0.993	0.001
DLco % predicted	1.003	0.995-1.010	0.495
Total lung capacity	1.007	0.992-1.022	0.351
Vital capacity	0.994	0.985-1.002	0.155
Inspiratory capacity	0.993	0.987-0.999	0.034
Functional residual capacity	1.009	1.003-1.016	0.005
Residual volume	1.007	1.002-1.012	0.003
COPD assessment test	1.051	1.032-1.071	0.000
SGRQ	1.024	1.015-1.032	0.000
6-minute walk distance	0.998	0.997-0.999	0.005
White blood cells	1.135	1.063-1.213	0.000

Red blood cells	0.864	0.647-1.151	0.318
Hemoglobin, g/dL	0.921	0.836-1.013	0.089
Hematocrit, %	0.974	0.944-1.006	0.110
Platelet	1.002	1.000-1.004	0.066
Erythrocyte sedimentation rate	1.017	1.007-1.028	0.001
Neutrophils	1.012	0.998-1.026	0.085
Lymphocytes	0.989	0.973-1.005	0.191
Monocytes	0.873	0.814-0.933	0.000
Eosinophils	1.044	0.995-1.095	0.077
Basophils	0.838	0.578-1.179	0.329
Albumin	0.536	0.362-0.790	0.002
Blood urea nitrogen	0.991	0.964-1.018	0.529
Immunoglobulin E	1.000	0.999-1.001	0.645
C-reactive protein	1.047	0.995-1.117	0.125
Fibrinogen	1.004	1.002-1.007	0.001
D-dimer	1.620	1.151-2.408	0.012
Fractional exhaled nitric oxide	1.012	0.986-1.037	0.350
Myocardial infarction	1.809	0.974-3.326	0.057
Heart failure	0.448	0.149-1.1	0.108
Peripheral vascular disease	0.963	0.335-2.462	0.939
Diabetes mellitus	0.774	0.536-1.103	0.163
Hypertension	0.962	0.727-1.271	0.785
Osteoporosis	2.713	1.412-5.288	0.003
Gastroesophageal reflux disease	1.449	1.001-2.085	0.047
Hyperlipidemia	0.752	0.494-1.124	0.174
Thyroid disease	0.694	0.271-1.577	0.409
Inflammatory bowel disease	0.420	0.022-2.618	0.429

COPD, chronic obstructive pulmonary disease; FEV_1, forced expiratory volume in one second; FVC, forced vita capacity; DLco, carbon monoxide diffusing capacity; SGRQ, St. George's Respiratory Questionnaire.
